# Chicken interferon-induced transmembrane proteins inhibit Newcastle disease virus infection by affecting viral entry and W protein expression

**DOI:** 10.1186/s13567-025-01530-y

**Published:** 2025-05-21

**Authors:** Jing Chen, Peiheng Li, Wancheng Zou, Ju Li, Yuhang Jiang, Letian Li, Pengfei Hao, Zihan Gao, Jiayi Hao, Xiaoshuang Shi, Chang Li

**Affiliations:** 1 State Key Laboratory for Diagnosis and Treatment of Severe Zoonotic Infectious Diseases, Key Laboratory of Zoonosis Research, Ministry of Education, College of Basic Medical Science, Jilin University, Changchun, 130012 China; 2 Research Unit of Key Technologies for Prevention and Control of Virus Zoonoses, Chinese Academy of Medical Sciences, Changchun Veterinary Research Institute, Chinese Academy of Agricultural Sciences, Changchun, 130122 China

**Keywords:** Chicken, IFITMs, NDV, virus entry, W protein

## Abstract

Interferon-induced transmembrane proteins (IFITMs) are essential components of the innate immune system, demonstrating potent resistance to various enveloped viruses (such as influenza, West Nile, and dengue viruses) both in laboratory settings and in living organisms. Newcastle disease (ND), resulting from Newcastle disease virus (NDV), is a severe avian viral ailment with notable economic impact due to its significant mortality and morbidity rates. On the basis of the efficient antiviral effects of IFITMs, an in-depth study of the role and mechanism of NDV inhibition by chicken IFITMs (chIFITMs) is highly important for the prevention and control of this disease. In this study, we found that transient overexpression of chIFITMs effectively inhibited NDV (NDV Lasota, NDV Na) infection in DF-1 cells, with the highest inhibition rates of up to 89% and 99%, respectively, and that there was no significant difference in the antiviral effects of chIFITM1/2/3, which were not significantly different. Virus‒cell binding-entry assays revealed that chIFITMs restrict the entry process of NDV. Deleting endogenous chIFITMs enhances viral replication (more than 1.27-fold) and diminishes chIFNL3-mediated antiviral effects. Concurrently, overexpressing chIFITMs influences the expression level of the W protein; and co-immunoprecipitation experiments confirmed interaction between them. These findings suggest that the W protein could represent a novel target for the inhibition of NDV by chIFITMs. In summary, our results provide the initial comprehensive analysis of the antiviral effects of chIFITMs against NDV. This observation suggests that IFITMs are important barriers against zoonotic infections and important targets against viral invasion.

**Supplementary Information:**

The online version contains supplementary material available at 10.1186/s13567-025-01530-y.

## Introduction

Interferon-induced transmembrane proteins (IFITMs) are small transmembrane proteins activated by interferon (IFN). These proteins have gained significant attention in antiviral research because of their widespread antiviral capabilities and unique ability to impede viral invasion [1]. Since they were first shown to increase the natural resistance of cells to viral infection in 1996 [2], IFITMs have been shown to restrict various viral infections, including major pathogens such as influenza A virus (IAV), Ebola virus (EBOV), severe acute respiratory syndrome coronavirus 2 (SARS-CoV-2), human immunodeficiency virus (HIV), and Zika virus (ZIKV) [3–9]. In addition to human IFITMs (huIFITMs), studies on IFITMs from mice, pigs, chickens, and ducks revealed their effectiveness in blocking influenza virus replication [10–15].

IFITMs physically alter cell membrane properties, and IFITMs (especially IFITM3) embed themselves in the host cell membrane or endosomal membrane and alter the physical properties of the membrane (e.g., increasing rigidity and changing curvature) through their transmembrane structural domains [16]. These changes directly impede the fusion of the viral envelope with the host membrane, which is particularly effective for membrane fusion-dependent enveloped viruses (e.g., influenza virus, HIV, and SARS-CoV-2) [5]. IFITMs also inhibit the aggregation and function of viral envelope proteins by modulating the distribution of cholesterol and sphingolipids in the cell membrane and destroying the structure of lipid rafts; they also block viral endosomal escape, which is important for viruses (e.g., influenza virus, EVD, and SARS-CoV-2) that enter the cell membrane through the endocytosis route. For viruses that enter through the endocytosis route (e.g., influenza virus, Ebola virus), IFITM3 is enriched in endosomal membranes (e.g., late endosomes/lysosomes) and prevents the fusion of the viral envelope and endosomal membranes by altering the pH or cholesterol distribution in the lumen of the endosomes, preventing the viruses from releasing their genetic material [17]. For example, IFITM3 prevents the conformational change of the influenza virus hemagglutinin (HA) protein in a low-pH environment, preventing it from initiating membrane fusion; IFITMs directly interact with viral components, and IFITMs may directly bind to viral envelope proteins [18] (e.g., gp41 of HIV, the E protein of dengue virus), interfering with their membrane fusion-mediated function; and IFITMs interfere with the membrane fusion function mediated by binding to viral replication IFITMs indirectly inhibit viral replication by binding to host factors required for viral replication (e.g., the cholesterol transporter protein VAPA [19]). IFITMs regulate cellular cholesterol metabolism, and by interfering with cellular cholesterol transport (e.g., by inhibiting the function of the NPC1 protein), IFITM3 leads to the accumulation of cholesterol abnormally in endosomes, resulting in the redistribution of cholesterol and the disruption of the lipid environment necessary for virus fusion. Certain viruses (e.g., SARS-CoV-2) rely on host cholesterol to complete their invasion, and IFITMs block this process by decreasing membrane cholesterol availability and inhibiting cholesterol utilization by viruses [20]. In conclusion, IFITMs form a broad-spectrum antiviral barrier through multiple mechanisms, including physical alteration of host membrane properties, blockade of viral membrane fusion, regulation of cholesterol metabolism and direct interactions. Their effects are highly dependent on subcellular localization and viral invasion pathways, and they are key molecules linking natural immunity to host restriction factors. An in-depth study of the mechanisms of IFITMs could provide important targets for the development of novel antiviral drugs, such as compounds that mimic their membrane-modifying functions. IFITMs may play a crucial role in disease control. For example, chicken flocks exhibiting high chicken IFITM (chIFITM) expression in intensive breeding environments could reduce virus transmission and contain outbreaks. Screening for chicken breeds with high chIFITM expression or specific genotypes, such as single-nucleotide polymorphisms, holds genetic breeding potential for developing disease-resistant lines and decreasing antibiotic reliance. Additionally, by enhancing natural antiviral capabilities, avian mortality and production losses can be reduced, particularly in the prevention and control of highly lethal diseases such as avian influenza and infectious bursal disease.

Chicken and duck IFITMs have relatively high amino acid homology and relatively close affinities, and chicken IFITM2, IFITM3 and duck IFITM3 exhibit antiviral activity against various influenza virus subtypes [10].

Newcastle disease (ND) is a highly pathogenic avian viral disease with economic implications due to elevated mortality and morbidity rates [21]. While much research on Newcastle disease virus (NDV) has focused on virus evolution and vaccine development [22–25], few studies have explored virus‒host interactions and how hosts employ innate immunity to restrain NDV replication [26, 27]. The NDV genome encodes a variety of structural and non-structural proteins that play key roles in viral replication, host immune escape, and pathogenicity. Fusion proteins (F proteins) mediate the fusion of the viral envelope with host cell membranes, a critical step in viral invasion. The sequence of the cleavage site of F proteins determines virulence, and the cleavage site of strong strains is readily recognized by a wide range of host proteases, which may be relevant to mammalian infections. Recombinant F proteins or viral vector vaccines expressing F proteins (e.g., those based on a weak strain of LaSota) have been widely used for avian immunization [28, 29]. Hemagglutinin-neuraminidase (HN protein) recognizes salivary acid receptors on the host cell surface, promotes viral attachment and prevents viral aggregation through neuraminidase activity. Monoclonal antibodies against HN proteins inhibit a wide range of NDV strains, providing a new strategy for general-purpose vaccine development [30, 31]. Matrix proteins (M proteins) regulate viral assembly and budding and maintain the morphology of viral particles. Some studies have shown that M proteins promote viral release by binding to the host ESCRT complex, and at the same time, M proteins can inhibit the host NF-κB pathway, which can weaken inflammatory responses [32]. Nucleocapsid proteins (NPs) encapsulate viral RNA to form nucleocapsids, which protect the genome and participate in transcription/replication, and they are used as antigens in ELISA for rapid diagnosis. ELISA can be used for the rapid diagnosis of NDV infection and the assembly of VLPs using NP and F/HN proteins as vaccine candidates without genetic material [33, 34]. The non-structural protein V protein inhibits innate immune signalling and antagonizes the host IFN response by binding to molecules such as MDA5/STAT1, and the NDV strains in which the V protein was knocked down by CRISPR presented an attenuated phenotype in a mouse model, suggesting that its potential as a live attenuated vaccine [26, 35]. The function of the W protein is not fully defined and may be related to the regulation of the viral replication cycle and that the cellular localization of the W protein affects NDV virulence [36]. Despite the well-documented antiviral efficacy of IFITMs from diverse species, it remains uncertain whether chIFITMs possess antiviral activity against NDV. As a core component of chicken innate immunity, chIFITM provides a new strategy for disease prevention and control through the dual mechanism of direct antiviral and immunomodulation.

This study revealed that chIFITMs effectively hindered the replication of diverse viruses, including NDV, vesicular stomatitis virus (VSV), and H9N2 IAV, in chicken fibroblasts. Notably, these compounds increased the survival rate of virus-infected cells. Depleting endogenous chIFITMs had the opposite effect, promoting viral proliferation and diminishing the antiviral activity of chicken interferon lambda 3 (chIFNL3). Further investigations demonstrated that chIFITMs impeded viral entry and could reduce the expression levels of W proteins, directly interacting with these proteins.

In summary, our results analysed the antiviral properties of chIFITMs, emphasizing their importance in the interferon pathway. These proteins play crucial roles in inhibiting the early entry of NDV and impacting the virulence protein W. The findings of this study offer valuable insights for the prevention and treatment of other viruses belonging to the *Paramyxoviridae* family.

## Materials and methods

### Cell lines

Chicken fibroblasts (DF-1) and chicken primary fibroblasts (CEF) were maintained in Dulbecco’s modified Eagle’s medium (DMEM; HyClone, USA) enriched with 10% foetal bovine serum (FBS; Gibco, USA) and 1% penicillin/streptomycin (Cytiva, USA). Culturing was performed in a humidified environment with 5% CO_2_/95% air at 37 °C. DF-1 cells were purchased from ATCC, and CEF cells were primary cells isolated from chicken embryos in our laboratory. DF-1 cells are third-generation cells at the time of use, whereas CEFs are first-generation cells. Both were used mainly as cell lines for in vitro antiviral phenotypic validation, which facilitated the analysis of changes in chicken-derived related proteins.

### Reagents

Lipofectamine 3000 Transfection Reagent and Lipofectamine™ RNAiMAX Transfection Reagent were purchased from Invitrogen (Carlsbad, CA, USA). TRIzol reagent was purchased from Sangon Biotech (Shanghai, China). M-MLV Reverse Transcriptase RNase and GoTaq^®^ were purchased from Promega (Madison, WI, USA). HRP-labelled goat anti-rabbit IgG (H + L) was purchased from Beyotime (Shanghai, China). Pierce ECL Western Blotting Substrate was purchased from Thermo Scientific (Waltham, MA, USA) [37].

### Viruses

The velogenic NDV strain Na (NDV-Na-EGFP; GenBank No. DQ659677.1) was obtained from Prof. Zhuang Ding, Jilin University, China. The attenuated NDV strain (NDV-rL-EGFP) [38] and the IAV strain H9N2 (A/Chicken/Guangdong/SS/1994; GenBank No. DQ874395.1) were provided by Prof. Ming Liao, South China Agricultural University, China. Both NDV and IAV were propagated in embryonated chicken eggs. Viral titres were measured in DF-1 cells using the 50% tissue culture infective dose (TCID_50_) method. The VSV strains with an enhanced green fluorescent protein-encoding gene (VSV-EGFP; a gift from Prof. ZhiGao Bu, Harbin Veterinary Research Institute, China) [39] were also titrated on BHK-21 cells by TCID_50_ [40].

### TCID_50_

The cells were seeded into 96-well plates at 1 × 10^5^ cells per well, and the virus sample was diluted tenfold to 100 μL/well (3 repetitions for each sample). After the cells had obvious cytopathogenic effects (CPE), the number of CPE wells under each dilution was recorded, and we defaulted to positive wells when more than 50% of the cells in the wells were diseased. The TCID_50_ was calculated via the Reed‒Muench method [41].

### Gene amplification and bioinformatics analysis

cDNA sequences of chIFITM1 (GenBank No. NM001350059.2), chIFITM2 (GenBank No. NM001350058.2), and chIFITM3 (GenBank No. NM001350061.2) were synthesized from primary DF-1 cells via reverse transcription‒polymerase chain reaction (RT‒PCR) with specific primers. Additionally, the W gene was amplified from cDNA obtained from NDV/Na virus-infected DF-1 cells.

The specific primers used were as follows:

chIFITM1 gene, 5ʹAGCTTGCCACCATGCAGAGCTACCCTCAGCACACCA-3ʹ (forward, chIFITM1F) and 5ʹ-GGTACCTCAAGCGTAGTCTGGGACGTCGTATGGGTAGGGCCGCACAGTGTACAACGG-3ʹ (reverse, chIFITM1R); chIFITM2 gene, 5ʹ AAGCTTGCCACCATGAAGCCGCAACAGGCGGAGGTGA-3ʹ (forward, chIFITM2F) and 5ʹ-GGTACCCTAAGCGTAGTCTGGGACGTCGTATGGGTATCTGCTGATCGCGGTGATG-3ʹ (reverse, chIFITM2R); chIFITM3 gene, 5ʹ-AAGCTTGCCACCATGGAGCGGGTACGCGCTTCGGGTC-3ʹ (forward, chIFITM3F) and 5ʹ-GGTACCCTAAGCGTAGTCTGGGACGTCGTATGGGTAAGTGGGTCCAATGAATTCGGG-3ʹ (reverse, chIFITM3R); W gene, 5ʹ-GCTAGCATGCATCATCACCATCACCATATGGCCACTTTTACAGATGCAGAG-3ʹ (forward, WF) and 5ʹ-GAATTCTTACATCGCCTGCGCAAAGTCGGCAGGTAGCTGGACACGA-3ʹ (reverse, WR); The IFITM sequences from different species were acquired from NCBI. The phylogenetic analysis was performed using the maximum likelihood method with a bootstrap value of *n* = 1000 in the MEGA program version 7. The three-dimensional structure of IFITM was determined using AlphaFold. Alignment analysis was performed using the DNAstar program (DNASTAR Inc., USA).

### Plasmid construction and transfection

All three genes were subcloned and inserted into the pcDNA3.1 eukaryotic expression vector (Invitrogen, USA), each featuring an HA tag at their C-terminal end (Figure 2A). The integrity of the target genes was confirmed through DNA sequencing by Comate Bioscience Co. Ltd., China. The cells were seeded in 6/12-well plates at a density of 5/1 × 10^5^ cells/well overnight to reach 70–80% confluency. Then, the cells were transfected with 4 μg of the plasmids using Lipofectamine 3000 or 50 μM siRNAs targeting IFITMs using Lipofectamine RNAiMAX Reagent for 48 h. After 48 h, the cells were collected and lysed using radioimmunoprecipitation assay (RIPA) buffer (Beyotime, China) supplemented with phenylmethanesulfonyl fluoride (PMSF, Beyotime, China). After transient transfection of chIFITM1/2/3, the expression of the target gene increased 4000-, 800-, and 10 000-fold, respectively; the knockdown effect was greater than 90%.

### CCK8 assay

Cell viability was assessed using the CCK-8 method (Dojindo, Japan) following the manufacturer’s instructions. The cells were seeded in 96/12-well plates at a density of 1/5 × 10^5^ cells/well in 100 μL of medium and cultured for 24 h. After treatment, 10/50 μL of sterile CCK-8 solution was added to each well and incubated for an additional 2 h at 37 °C. The absorbance at 450 nm was measured with a TECAN SPAPK microplate reader.

### Viral infection

DF-1 cells seeded at 5 × 10^5^ cells/well in 12-well plates were infected with the designated viruses at the appropriate MOIs (VSV-EGFP: 0.01 MOI; NDV-Na-EGFP: 1 MOI; NDV-rL-EGFP: 2 MOI; IAV strains H9N2: 5 MOI) (Additional file 2). Vector expression protein was used as a negative control.

### Virus adsorption assay

DF-1 cells stably expressing chIFITMs were infected with the virus at 4 °C for 1 h. After this period, the supernatant was removed, and the cells were washed three times with cold PBS to remove unadsorbed virus. The cells were then harvested, and the virus adsorption levels were quantified via qRT‒PCR [42].

### Virus entry assay

The procedure for the virus entry assay was similar to that for the virus adsorption assay. After the unadsorbed virus was washed off, prewarmed medium at 37 °C was added to the cells, and the mixture was incubated for another hour to facilitate virus entry [42].

### Fluorescence observation

After virus inoculation, the cells were examined for fluorescence every 12 h using a Thermo Fisher Scientific EVOS M5000 microscope.

### Western blot

Total cell extracts were prepared, resolved by 10% SDS‒PAGE, and then transferred onto PVDF membranes (GE Healthcare, Germany). The membranes were blocked with 5% skim milk and incubated with specific antibodies at room temperature for 2 h, followed by incubation with HRP-labelled goat anti-rabbit/mouse IgG (H + L) for 1 h. The protein bands were visualized using GEGEGNOME XRQ enhanced chemiluminescence (ECL) (Thermo Fisher Scientific, USA), with the antibodies listed in Table 1. In principle, the choice of internal reference is based on the difference in size from the target protein to select a common internal reference with a large difference in size (GAPDH, β-actin, or α-tubulin), and for the detection of cell nuclear proteins, the internal reference histone H3 is used.

**Table 1:** **Antibodies used in this study.**

Antibody	Manufacturer	Product No.	Dilutions
Anti-HA	Cell Signaling Technology (Boston, MA, USA)	5017S	1:1000
Anti-His	Cell Signaling Technology (Boston, MA, USA)	9991S	1:1000
Anti-GFP tag Monoclonal antibody	Proteintech (Chicago, USA)	66002-1-Ig	1:1000
Anti-Influenza A Virus HA	SinoBiological (Beijing, China)	86001-RM01	1:1000
W polyclonal antibody	Laboratory prepared polyclonal antibodies	–	1:1000
LC3 Polyclonal antibody	Proteintech (Chicago, USA)	14600-1-AP	1:1000
IFITM3 Polyclonal antibody	Proteintech (Chicago, USA)	11714-1-AP	1:1000
Anti-β-actin	Genetex (Shanghai, China)	GTX109639	1:1000
Anti-α-tubulin	Genetex (Shanghai, China)	GTX112141	1:1000
Anti-Histone H3	Cell Signaling Technology (Boston, MA, USA)	4499S	1:1000
Anti-mouse IgG (HRP-linked antibody)	Beyotime Biotechnology (Shanghai, China)	A0216	1:3000
Anti-rabbit IgG (HRP-linked antibody)	Beyotime Biotechnology (Shanghai, China)	A0208	1:3000

### RNA extraction

Total RNA was extracted from virus-infected cells or mock-treated cells using TRIzol Reagent according to the manufacturer’s instructions. The total RNA was dissolved in 50 μL of RNase-free ddH_2_O and stored at −20 °C [37].

### qRT‒PCR

Following the manufacturer’s guidelines, total RNA was extracted from cells using a kit from Sangon Biotech, China. The cDNA obtained was then analysed through quantitative PCR (qPCR) with Fast Start Universal SYBR Green Master Mix (Roche, USA). Relative gene expression levels were calculated and normalized against those of β-actin using the 2^−ΔΔCT^ method. The specific primers used for gene detection are as follows: IFITM1 gene, 5ʹ-GCCTGGGCTTATGTGCTCTC-3ʹ (forward) and 5ʹ-TGGGGGTGATACCAGAGGTAG-3ʹ (reverse); IFITM2 gene, 5ʹ-ATCTTCTCCATCAAGGCCCG-3ʹ (forward) and 5ʹ-ACAACACACCGACGGCTATC-3ʹ (reverse); IFITM3 gene, 5ʹ-GTGAAGTCCAGGGATCGCAA-3ʹ (forward) and 5ʹ-GGGTCCAATGAATTCGGGGT-3ʹ (reverse); NDV Na HN gene, 5ʹ-ATCCCGGCGCCTACTACAGGATCCGGTTGCACT-3ʹ (forward) and 5ʹ-ACTGCAGGACTTCCGATTTTGGGTGTCATCT-3ʹ (reverse); and β-actin gene, 5ʹ-TCCCGGCGCCTACTACAGGATCCGGTTGCACT-3ʹ (forward) and 5ʹ-ACTGCAGGACTTCCGATTTTGGGTGTCATCT-3ʹ (reverse).

Primer design was based on the principles of whole coding sequence amplification design with the primer design software Primer 5.0 (Premier, Canada).

### Construction of a cell line stably expressing chIFITMs

The coding sequences of the target genes chIFITM1, chIFITM2, and chIFITM3 were inserted into the eukaryotic expression vector pLV-TRE3G using standard molecular cloning techniques, resulting in the construction of the pLV-IFITM-HA plasmid, which features an HA tag at the C-terminus. DF-1 cells were generated through co-transfection with this constructed plasmid and the pLV-Tet3G plasmid to induce target gene expression [43]. Successfully recombined monoclonal cell lines were selected using G418 and puromycin. The expression of the target protein in the recombinant cells was verified by qRT‒PCR and western blotting, as detailed in Table 1.

In a separate process, chIFITM1, chIFITM2, and chIFITM3 were synthesized from primary DF-1 cells and amplified by reverse transcription‒polymerase chain reaction (RT‒PCR) with specific primers.

The specific primers used were as follows: pLV-chIFITM1 gene, 5ʹ-AAGCTTGCCACCATGCAGAGCTACCCTCAGCACACCA-3ʹ (forward, pLV-chIFITM1F) and 5ʹGGTAGAATTCCATATGTCAAGCGTAGTCTGGGACGTCGTATGGGTAGGGCCGCACAGTGTACAACGG-3ʹ (reverse, pLV-chIFITM1R); pLV-chIFITM2 gene, 5ʹ-GGATCCGCCACCATGAAGCCGCAACAGGCGGAG GTGA-3ʹ (forward, pLV-chIFITM2F) and 5ʹ-GAATTCCTAAGCGTAGTCTGGGACGTCGTAT GGGTATCTGCTGATCGCGGTGATG-3ʹ (reverse, pLV-chIFITM2R); pLV-chIFITM3 gene, 5ʹ-ACTTGGATCCGGGCCCGCCACCATGGAGCGGGTACGCGCTTCGGGTC-3ʹ (forward, pLV-chIFITM3F) and 5ʹ-GGTAGAATTCCATATGCTAAGCGTAGTCTGGGACGTCGTATGGGTAAGTGGGTCCAATGAATTCGGG-3ʹ.

### siRNA silencing

DF-1 cells were seeded in 6-well plates at a density of 1 × 10^5^ cells/well and cultured overnight to achieve 70–80% confluency. The cells were subsequently transfected with 50 nM siRNAs targeting IFITMs (RiboBio Co., Ltd., China), utilizing Lipofectamine RNAiMAX Reagent (Thermo Fisher Scientific, USA). This transfection was carried out for 48 h, in strict accordance with the manufacturer’s protocol [37, 44].

The sequences of the siRNAs used were as follows: siIFITM1-1, GGATCATCGCCAAGGACTT; siIFITM2-1, CGCTCATCTTCTCCATCAA; and siIFITM3-1, GCGAAGTACCTGAACATCA.

### Flow cytometry (FCM)

EGFP-positive cells were harvested at predetermined time intervals, suspended in PBS, and then visualized using fluorescence microscopy. For quantification, these cells were analysed using a CytoFLEX flow cytometer (Beckman Coulter) [41].

### Coimmunoprecipitation

For coimmunoprecipitation (Co-IP), DF-1 cells were co-transfected with HA-fused and His-fused protein expression plasmids for 48 h. After transfection, the cells were lysed using IP lysis buffer (Beyotime, China). The antigen sample was then incubated with 10 µg of either anti-HA or anti-His antibody. The reaction volume was adjusted to 500 µL with cell lysis buffer, and the mixture was incubated at room temperature for 1–2 h or mixed overnight at 4 °C. The diluted sample was added to a tube containing prewashed magnetic beads, followed by gentle vortexing or inversion to mix. This mixture was incubated at room temperature with mixing for 1 h. The beads were then collected using a magnetic stand, and the supernatant was removed and discarded. The beads were washed by adding 500 µL of binding/wash buffer, mixed well, and then collected with a magnetic stand, after which the supernatant was discarded. This washing step was repeated twice. Finally, 100 µL of SDS‒PAGE reducing sample buffer was added to the tube, and the sample was heated at 96–100 °C for 10 min in a heating block [45].

### Confocal microscopy

The cells were fixed in a 4% paraformaldehyde solution (Solarbio, China) for 1 h. Following fixation, they were washed three times and then blocked with BSA for 1 h. After another three washes, specific primary antibodies were added, and the cells were incubated at 4 °C overnight. The cells were subsequently washed again and incubated with either anti-rabbit or anti-mouse fluorescent secondary antibodies for 1 h. Finally, images of the cells were captured by fluorescence or confocal microscopy.

### Medication

3-MA (189490-50 MG) and MG132 (474790-1 MG) were purchased from MCE. 3-MA was diluted with DMSO to prepare a 1 M solution, which was subsequently diluted into the culture medium as needed. 3-MA was used at a concentration of 5 mM. Before the addition of MG132, the mixture was incubated in a water bath at 20–25 °C until it was completely dissolved. MG132 was diluted to 5 μM with DMSO according to the instructions. Twenty-four hours after co-transfection of the expression plasmid, the cells were treated with the proteasome inhibitor MG132 or the autophagy inhibitor 3-MA for 12 h.

### Statistical analyses

Statistical analysis was conducted using GraphPad 9.0 (GraphPad Software, San Diego, CA, USA). Comparisons between 2 groups were made by an unpaired t test. Comparisons among three or more groups were performed via multiple comparisons, and *P* values were derived from one-way ANOVA (Dunnett’s multiple comparisons test, 95% confidence intervals), adjusted to compare the mean of each column with that of a control column, and two-way ANOVA (Tukey’s multiple comparisons test, 95% confidence intervals). For all comparisons, *P* < 0.05 was considered statistically significant. Each distinct set of assays was evaluated in at least three independent experiments. The outcomes are presented as the means ± standard deviation (SD).

## Results

### Genomic architecture and genetic analysis of chIFITMs

**Figure 1:**
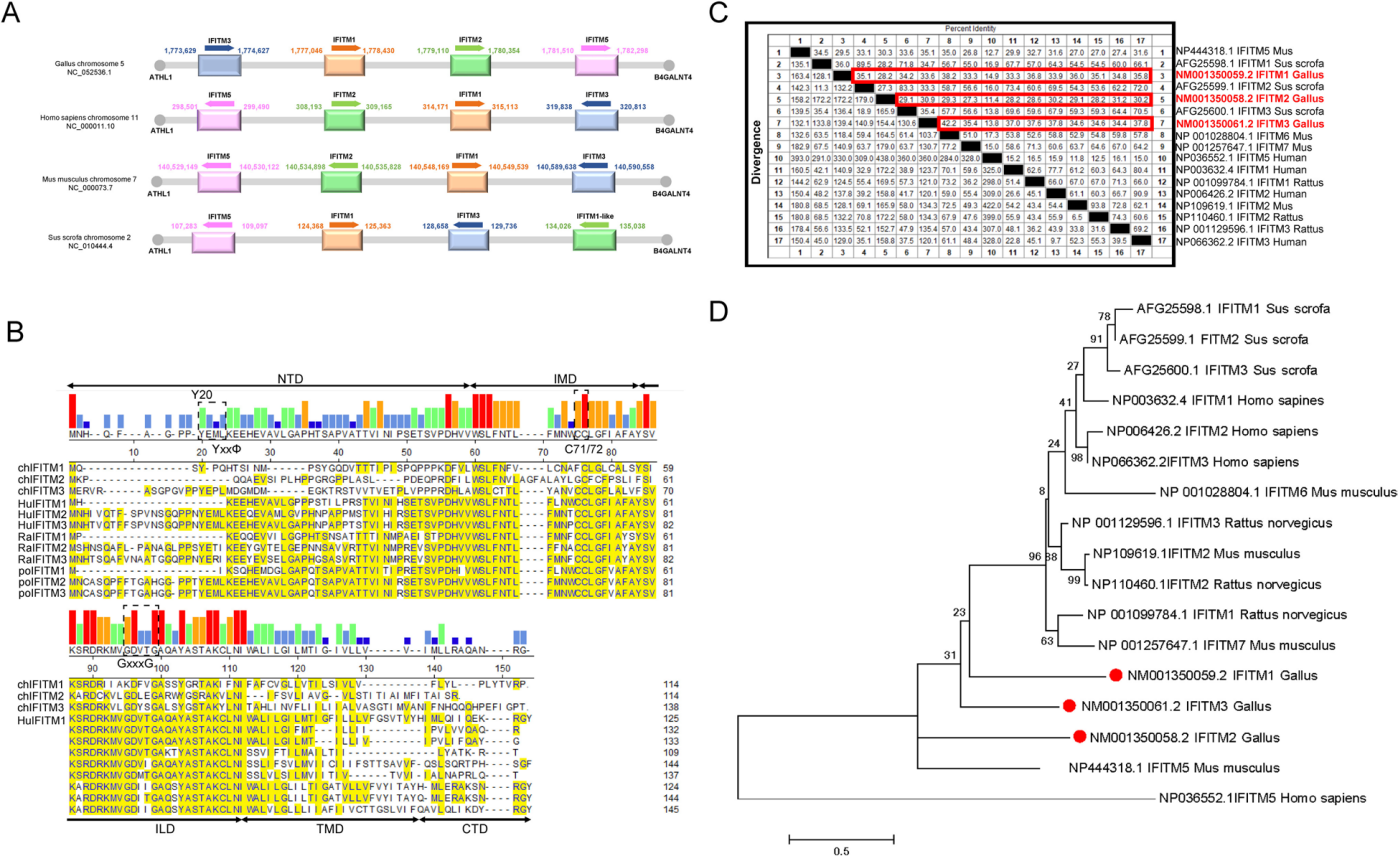
**Genomic architecture and genetic analysis of chIFITMs.** **A** Genomic locations of IFITMs in various species. **B** Amino acid alignment of chicken IFITM1, IFITM2, and IFITM3 with those from humans, rats, and pigs. **C** Homology comparison of chicken IFITMs (IFITM1, GenBank No. NM001350059.2; IFITM2, GenBank No. NM001350058.2; IFITM3, GenBank No. NM001350061.2) with those from other species. **D** Phylogenetic analysis of IFITMs across different species conducted using a maximum likelihood (ML) tree with a bootstrap value of *n* = 1000. The inset illustrates the phylogenetic relationships of IFITMs among various species.

The genomic structure and genetic analysis of chIFITMs revealed that the chIFITM family, comprising IFITM 1, 2, -3, and -5, is located on chromosome 5 in chickens. This locus is located between the centromeric acidic dendrimer-like 1 (ATHL1) gene and the telomeric -1,4-N-acetyl-galactosaminyltransferase 4 (B4GALNT4) gene [46]. Notably, the chIFITM genes occupy a similar locus as their counterparts in humans, mice, and pigs (Figure 1A). Sequence comparisons between IFITMs from various species and chIFITMs revealed that chIFITMs share similar structural and functional loci. The key features include Y (Y20) [47], C71/72 [48], a motif (GxxxG) [6], and YxxΦ [49] (Figure 1B). Homology analysis revealed that the chIFITMs and IFITMs from all the species presented less than 50% similarity (Figure 1C). Furthermore, genetic evolutionary studies demonstrated that chIFITMs form a distinct branch separate from mammals (Figure 1D). This finding suggests significant differences between chIFITMs and mammalian IFITMs, despite sharing significant sites and motifs. These differences suggest potentially unique phenotypes and mechanisms in chIFITMs.

### Transient overexpression of chIFITMs inhibits the proliferation of NDV and other viruses

**Figure 2:**
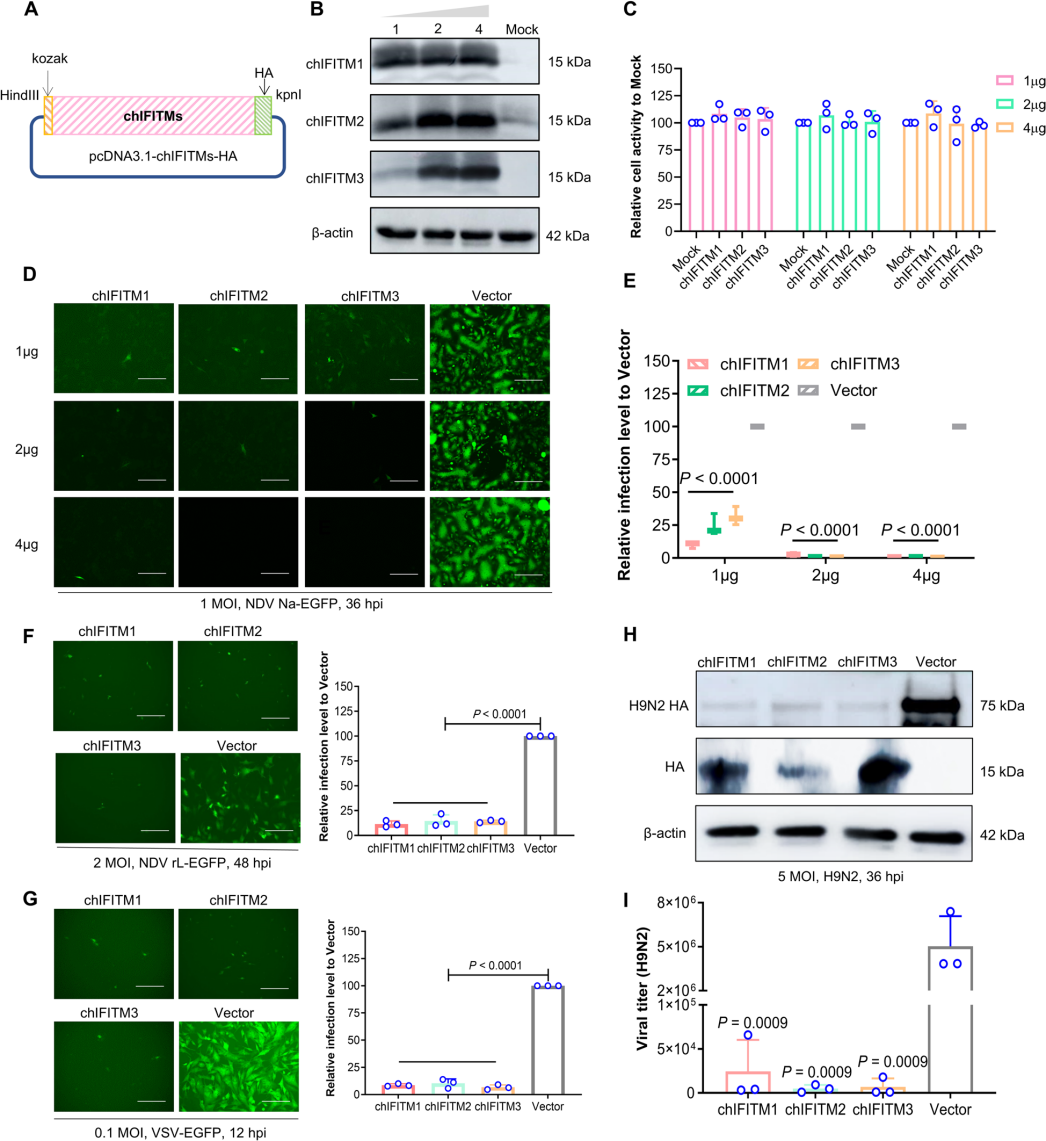
**Antiviral activity of chIFITMs.** **A** Diagrams of the engineered plasmid pcDNA3.1-chIFITMs, with primers detailed in the Materials and methods section. **B** Western blotting was used to detect the expression of chIFITM (overexpression doses of 1, 2, and 4 μg). HA/β-actin-specific antibodies were used as primary antibodies, and HRP-labelled goat anti-rabbit/mouse IgG (H + L) was used as the secondary antibody. **C** Toxic effects of different doses of chIFITM on cells were assessed by CCK8 analysis. **D**–**G** Evaluation of the antiviral efficacy of chIFITM via fluorescence observation and flow cytometry in 12-well plates at 36 h post-infection with NDV Na-EGFP (1 MOI) (**D**, **E**) and at 36 h post-infection with VSV-EGFP (0.1 MOI) and NDV rL-EGFP (2 MOI) (**F**, **G**); an empty vector was used as a negative control. (H-I) Investigation of the antiviral effects of chIFITMs by western blot and viral titre assays at 36 h after H9N2 (MOI of 5) infection; the empty vector was used as a negative control. The scale bar represents 150 μm. Significance levels are indicated as follows: *P < 0.05; **P < 0.01; ***P < 0.001; ****P < 0.0001; ns, no significant difference.

To evaluate the antiviral effectiveness of chIFITMs (chIFITM1, 2, 3), plasmids containing chIFITMs were engineered with the pcDNA3.1 vector (Figure 2A). DF-1 cells were transfected with these constructs, while an empty vector served as the negative control. The results confirmed the correct expression of chIFITMs (Figure 2B) and indicated that overexpression of IFITMs did not impair cell activity (Figure 2C). Antiviral testing revealed that chIFITMs suppressed NDV Na virus infection in a dose-dependent manner, achieving inhibition rates between 50 and 95%. Transfection of different chIFITMs at 1 μg resulted in greater restriction by chIFITM1, followed by chIFITM2 and chIFITM3, and there were no significant differences in the antiviral effects among chIFITM1, chIFITM2, and chIFITM3 at 2 μg and 4 μg (Figures 2D, E). Further experimentation demonstrated that chIFITMs effectively inhibited the proliferation of NDV rL-EGFP and VSV-EGFP (NDV rL-EGFP at an MOI of 2, VSV-EGFP at an MOI of 0.1), with inhibition rates of 90% and 85%, respectively (Figures 2F, G). Additionally, the overexpression of chIFITMs reduced the expression level of the H9N2 HA protein (H9N2 AIV at an MOI of 5) and significantly reduced the viral titre of H9N2 by more than 100-fold (Figures 2H, I).

### Depletion of chIFITMs modulates the anti-NDV effect mediated by IFN

**Figure 3:**
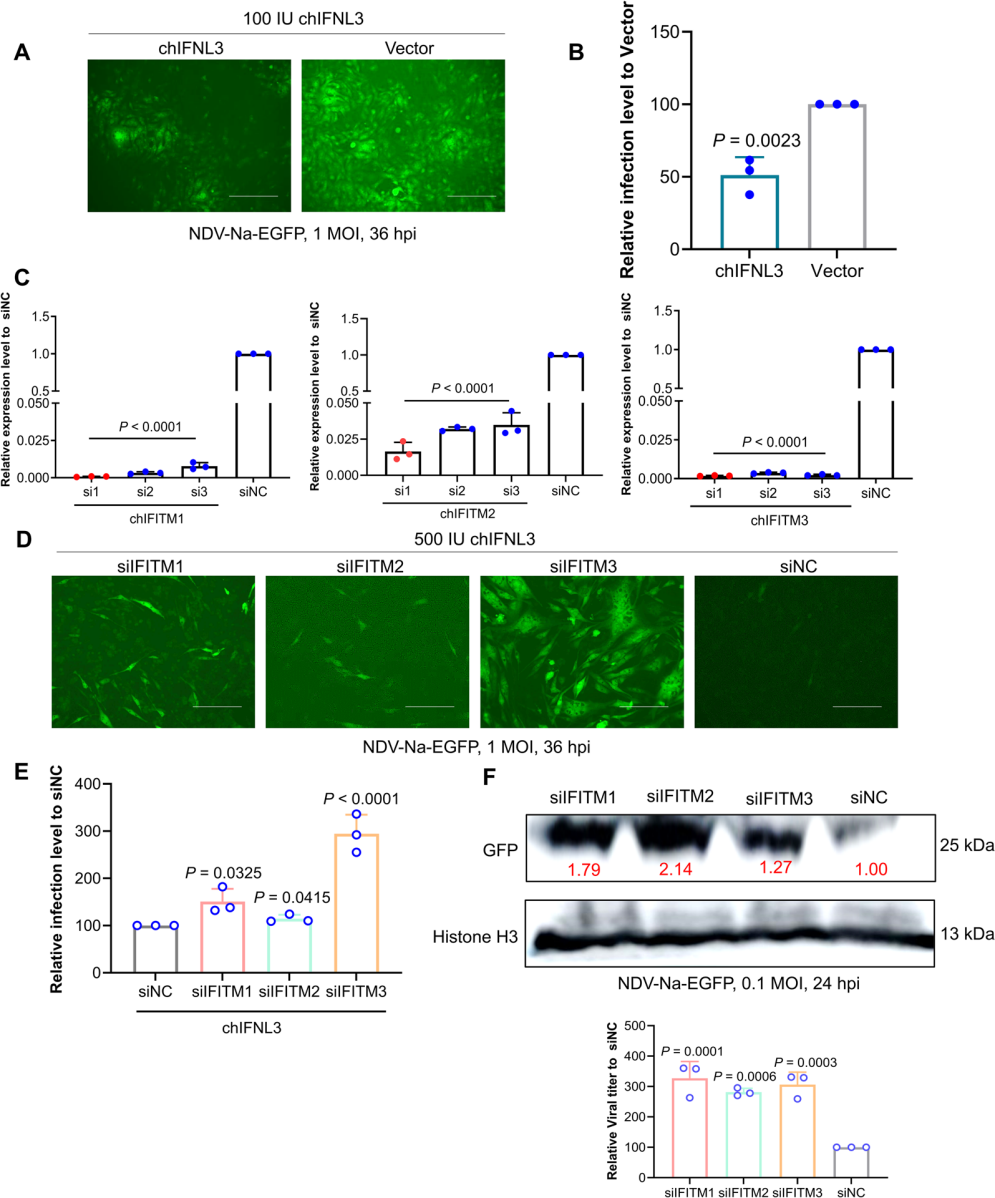
**Impact of chIFITM depletion on IFN-mediated anti-NDV activity.** **A**, **B** The antiviral effect of chIFNL3 was assessed via fluorescence and flow cytometry in 12-well plates 36 h after NDV Na-EGFP infection. A total of 100 IU chIFNL3 was used to pretreat the cells 12 h after virus infection. **C** siIFITMs were screened via qRT‒PCR. The knockdown efficiency was greater than 90%. **D**, **E** The antiviral impact of chIFNL3 after the knockdown of IFITM1, IFITM2, and IFITM3 was explored. DF-1 cells transfected with chIFITM1, 2, or 3 siRNA were treated with 500 IU chIFNL3 for 12 h, followed by NDV Na-EGFP infection at an MOI of 1. The antiviral effect was analysed through fluorescence and flow cytometry at 36 h post-infection. **F** Investigating the impact of viral infection post-chIFITM depletion. CEK cells transfected with chIFITM1, 2, or 3 siRNA were infected with NDV Na-EGFP (MOI of 0.1). GFP expression levels were analysed by western blot at 24 h post-infection, and the supernatant TCID_50_ was determined. The multiplicity of the experimental group (siIFITMs) compared with the control group (siNC) is shown in red.

To explore the role of chIFITMs in the IFN pathway, chicken interferon lambda 3 (chIFNL3) was used for analysis (40). chIFNL3, a member of the chicken IFN III family, regulates IFN-stimulated genes (ISGs) through the III IFN receptor, which encodes antiviral proteins such as IFITM and exerts antiviral effects through different mechanisms of action. The findings indicated that chIFNL3 significantly curtailed NDV Na-EGFP infection by approximately 50% (Figures 3A, B). The most effective small interfering RNAs (siRNAs) that target chIFITM 1, 2, and 3 were subsequently identified (Figure 3C, Additional file 1). This study then assessed how the silencing of chIFITMs impacts the antiviral efficacy of chIFNL3. The results revealed a variable reduction in the antiviral ability of chIFNL3, with siIFITM3 resulting in the most pronounced decrease (threefold) (Figures 3D, E). Additionally, the removal of endogenous chIFITMs led to increased viral proliferation (Figure 3F), underscoring the crucial role of chIFITMs, particularly chIFITM3, in the interferon-mediated antiviral pathway.

### NDV replication is significantly restricted in cells stably expressing chIFITMs

**Figure 4:**
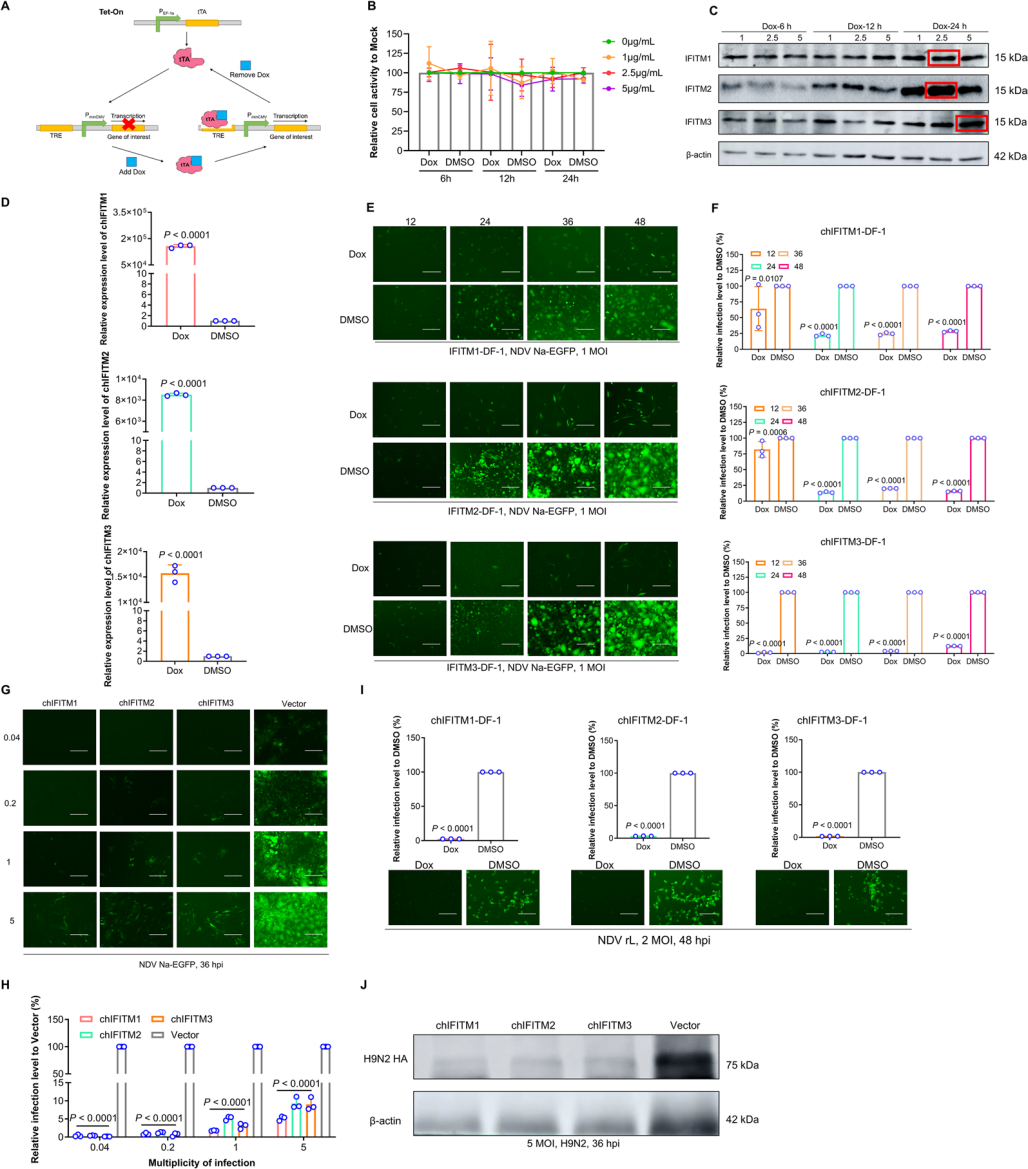
**Restricted NDV replication in cells stably expressing chIFITM.** **A** In accordance with the Tet-on system principle, the constructed successful cell lines were induced with Dox to express the target proteins; otherwise, they were not expressed. **B** CCK8 assay for assessing Dox and DMSO toxicity. Dox was the inducer, and DMSO was the dilution of the inducer, which was also used as a negative control. **C** Screening of the optimal inducer concentration and induction time and selection of optimal induction conditions according to the level of target protein expression under different conditions. chIFITM expression was confirmed by western blot using HA/β-actin primary antibodies and HRP-conjugated goat anti-rabbit/mouse IgG (H + L) secondary antibodies. The optimal induction conditions for chIFITM1/2/3 were 2.5 μg/mL for 24 h, 2.5 μg/mL for 24 h, and 5 μg/mL for 24 h. **D** qRT‒PCR analysis. The expression levels of chIFITM genes under optimal induction conditions increased approximately 100 000-, 8000-, and 10 000-fold, respectively. **E**, **F** Characterization of the antiviral capacity of the cell lines after induction for different durations at the optimal induction dose. Fluorescence observation and flow cytometry analysis of antiviral agents. **G**, **H**. Role of cell lines under optimal induction conditions against virus infections caused by different infectious complexes (0.04, 0.2, 1, and 5 MOIs). Fluorescence observation and flow cytometry analysis of antiviral agents. **I** Analysis of the antiviral role of cell lines against the weakly virulent NDV strain Lasota (NDV rL-EGFP) under optimal induction conditions. Fluorescence observation and flow cytometry analysis of antiviral agents. **J** Analysis of the antiviral role of the cell lines against H9N2; scale bar represents 150 μm. Significance levels are indicated as *P < 0.05; **P < 0.01; ***P < 0.001; ****P < 0.0001; ns, no significant difference.

To further verify and explore the antiviral mechanisms of chIFITMs, inducible DF-1 cell lines stably expressing chIFITMs were established with the Tet-on system, which consists of a regulatory expression vector and a reactive expression vector. The regulatory expression vector contains a human cytomegalovirus early promoter (PhCMV) and a reverse tetracycline-controlled transactivator (rtTA). The expression vector consists of a Tet-responsive element (TRE), a minimal CMV promoter (PminCMV) and the target gene. Since PminCMV lacks an enhancer, the target gene is not expressed when rtTA does not bind to the TRE; when rtTA binds to the TRE, VP16 activates PminCMV and results in gene expression. In the absence of Dox, rTetR cannot bind to TRE, resulting in the inhibition of gene expression; however, in the presence of Dox, rTetR can bind to TRE, which in turn results in the expression of the target gene (Figure 4A). Doxycycline (Dox) had no toxicity to the cells at the tested concentrations (Figure 4B). The optimal expression of chIFITM1/2/3 was achieved under Dox treatment at 2.5 μg for 24 h for chIFITM1 and chIFITM2 and at 5 μg for 24 h for chIFITM3 (Figure 4C). The results of quantitative PCR (qPCR) further verified that the induced cells expressed relatively high levels of the target genes (Figure 4D).

The antiviral capabilities of the positive cell lines were then assessed individually. Compared with the DMSO group, the Dox-induced group consistently inhibited NDV Na-EGFP infection at various time points. After 24–48 h of induction by Dox, the inhibition rates were all above 80%, the antiviral effect gradually increased with prolonged induction time, and a good antiviral effect was achieved after 24 h of induction, with no significant difference from that at 36 and 48 h of induction (Figures 4E, F), and at different multiplicities of infection (MOIs), there was greater than 85% inhibition in the high-dose infection group (Figures 4G, H). These strains also suppressed infections caused by NDV rL-EGFP (more than 95% inhibition rate) and H9N2 (Figures 4I, J). These findings aligned with those from the transient overexpression experiments, indicating the broad-spectrum antiviral effects of chIFITMs. These results suggest that the induced overexpression cell lines are suitable for further experimental studies.

### chIFITMs inhibit virus-induced cell death

**Figure 5:**
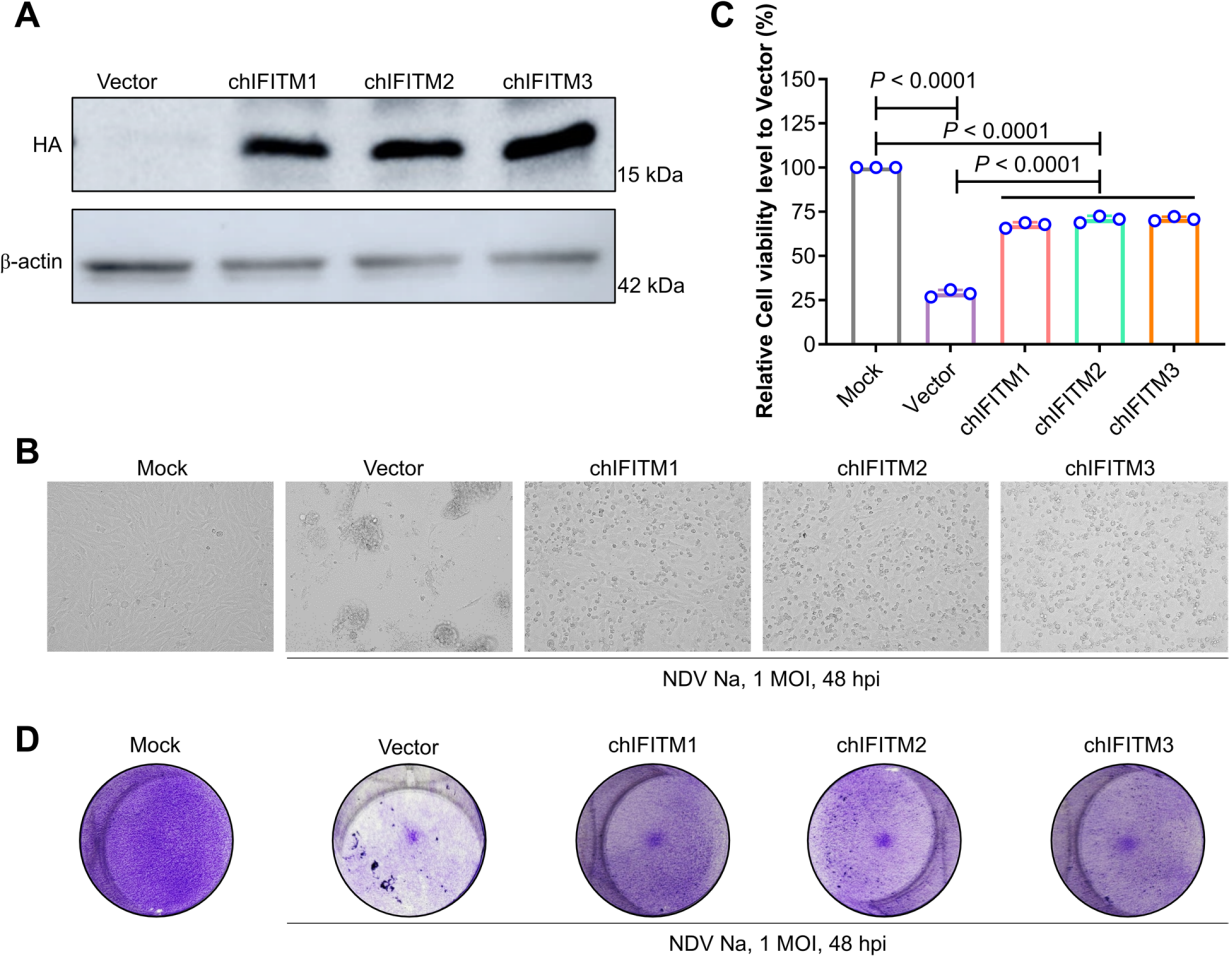
**chIFITMs prevent virus-induced cell death.** **A** chIFITM expression confirmed by western blot using HA/β-actin and HRP-conjugated goat anti-rabbit/mouse IgG (H + L) antibodies. **B** Cytopathic effects observed under a light microscope; scale bar, 150 μm. **C** Cell viability was analysed with a CCK8 assay; significance is indicated as follows: *P < 0.05; **P < 0.01; ***P < 0.001; ****P < 0.0001; ns, no significant difference. **D** Cell viability was further confirmed via crystal violet staining.

To examine the antiviral mechanisms of chIFITMs, an initial investigation focused on whether chIFITMs could prevent virus-induced cell death. The cytoprotective effects were analysed in cell lines overexpressing chIFITM1, 2, and 3 (Figure 5A). Observations revealed that the degree of cellular damage was less severe in the chIFITM group than in the control vector group (Figure 5B). A Cell Counting Kit-8 (CCK8) assay indicated that the overexpression of chIFITMs increased cell viability by 50% (Figure 5C). This finding was further corroborated by crystal violet staining (Figure 5D). Collectively, these data suggest that overexpressing chIFITMs reduces virus-induced cytopathic effects and enhances cell survival.

### chIFITMs suppress the early stages of NDV infection

**Figure 6:**
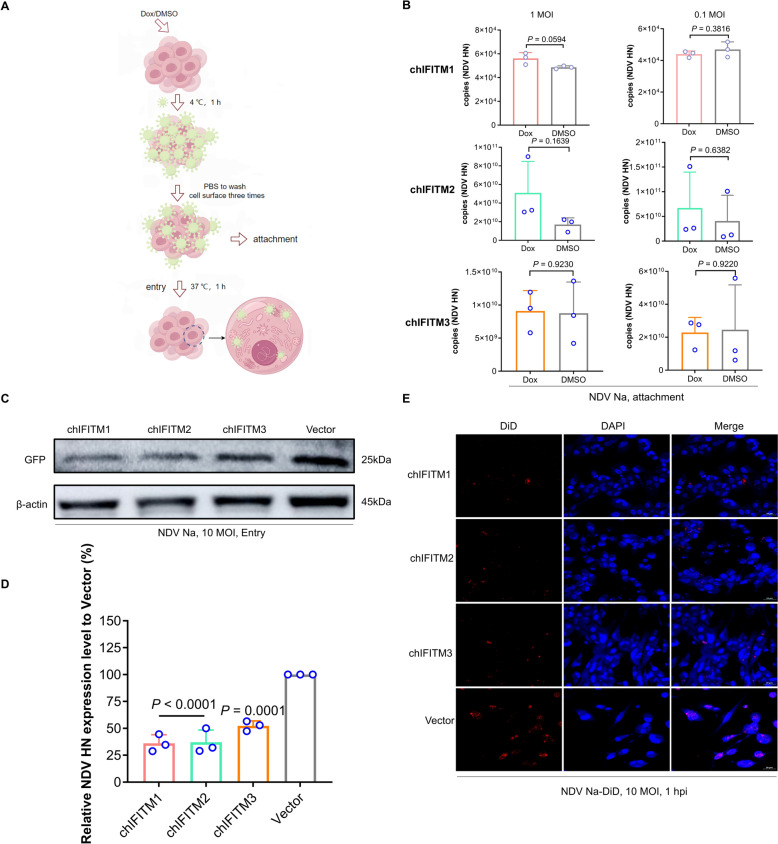
**chIFITMs inhibit early NDV replication stages by hindering viral entry.** **A** Diagram of the virus adsorption‒entry experiment. **B** Effects of the overexpression of chIFITMs at high and low infection multiplicities on the virus attachment process. qRT‒PCR analysis of the HN gene. **C** Effects of the overexpression of chIFITMs at high infection multiplicities on the virus entry process. GFP levels reflect viral entry. GFP levels were detected by western blot using GFP/β-actin primary antibodies and HRP-conjugated goat anti-rabbit/mouse IgG (H + L). **D** Effects of the overexpression of chIFITMs at high infection multiplicities on the virus entry process. NDV HN gene levels reflect viral entry. Additional qRT‒PCR assay for the HN gene. **E** DiD-labelled viruses are shown in red; increased red intensity corresponds to higher number of viruses entering the cells. Laser confocal microscopy for virus entry analysis, with significance levels indicated as *P < 0.05; **P < 0.01; ***P < 0.001; ****P < 0.0001; ns, no significant difference.

Classic virus‒cell adsorption and entry assays were conducted to determine whether chIFITMs could impede NDV attachment and entry (Figure 6A). The results of quantitative real-time PCR (qRT‒PCR) revealed no significant difference in the levels of the NDV HN protein between the chIFITM group and the control group. These findings suggest that chIFITMs do not affect virus adsorption to cells (Figure 6B). However, western blot and qPCR analyses revealed that the overexpression of chIFITMs significantly decreased the expression levels of both the GFP gene and the HN gene (Figures 6C, D). Interference with the GFP and HN expression indicates blockade of gene expression. To further validate the inhibitory effect of chIFITMs on viral entry, DF-1 cells overexpressing chIFITMs were infected with DiD-labelled NDV Na (red colour) and examined via laser confocal microscopy (Figure 6E). The findings demonstrated that, compared with vector control cells, chIFITMs presented lower levels of red fluorescence, indicating that chIFITMs reduce viral infection by hindering viral entry into cells.

### chIFITMs reduce the expression level of the NDV W protein

**Figure 7:**
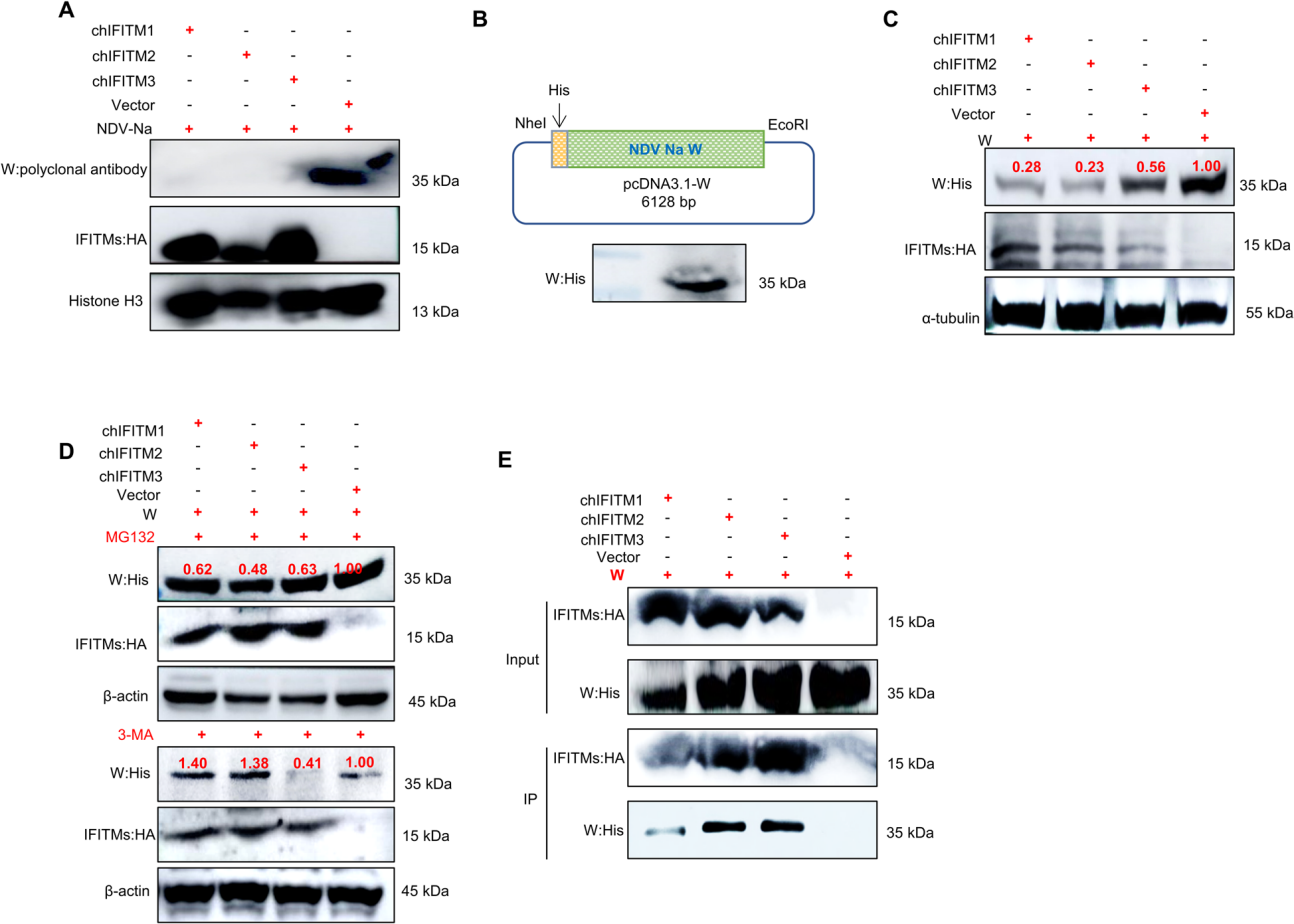
**chIFITMs reduce NDV W protein expression.** **A** NDV W protein expression was inhibited in chIFITMs by using a laboratory-prepared W polyclonal antibody and HA/Histone H3 primary antibodies with HRP-conjugated goat anti-rabbit/mouse IgG (H + L). **B** Schematic of pcDNA3.1-W recombinant plasmids and western blot confirmation of W protein expression. **C** Co-transfection of chIFITMS with the NDV W recombinant expression plasmid. Impact of chIFITM overexpression on W protein levels, as determined by His/HA/α-tubulin primary antibodies (W-His, chIFITM-HA) and HRP-conjugated goat anti-rabbit/mouse IgG (H + L). **D** Western blot analysis of the effects of the human proteasome inhibitor MG132 (5 μM) and the autophagy inhibitor 3-MA (5 mM) on chIFITM-mediated W protein inhibition. **E** Co-IP study of the interaction between chIFITMs and W protein.

The W protein is a key virulence factor of NDV, yet its characteristics are not well documented (36). Our study investigated the interaction between chIFITMs and the NDV W protein. Upon infecting cells overexpressing chIFITMs with NDV-Na, we observed a reduction in W protein expression (Figure 7A). To determine whether this decrease was due to inhibited viral entry, we constructed and expressed a eukaryotic plasmid containing the W gene (Figure 7B). The results demonstrated that, compared with those in the control group, the levels of W protein in chIFITMs were lower (Figure 7C), suggesting that chIFITMs themselves can suppress W protein expression. To further investigate the underlying mechanisms, we analysed two major protein degradation pathways: the proteasome degradation pathway and the autophagy degradation pathway. Following the administration of MG132, a proteasome inhibitor, the overexpression of chIFITMs continued to inhibit W protein expression. However, the application of 3-MA, an autophagy inhibitor, resulted in a significant increase in W protein levels in the chIFITM1 and chIFITM2 overexpression groups compared with those in the control group. Notably, chIFITM3 continued to impact W protein expression even after treatment with either MG132 or 3-MA, suggesting that chIFITM3 employs different methods to decrease W protein levels (Figure 7D). Co-IP results revealed a direct interaction between chIFITMs and the W protein (Additional file 3). This finding implies that chIFITMs might degrade the W protein through direct interaction (Figure 7E). However, further research is needed to fully understand the mechanisms involved.

### Preliminary study on the mechanism by which chIFITMs affect the action of W proteins

**Figure 8:**
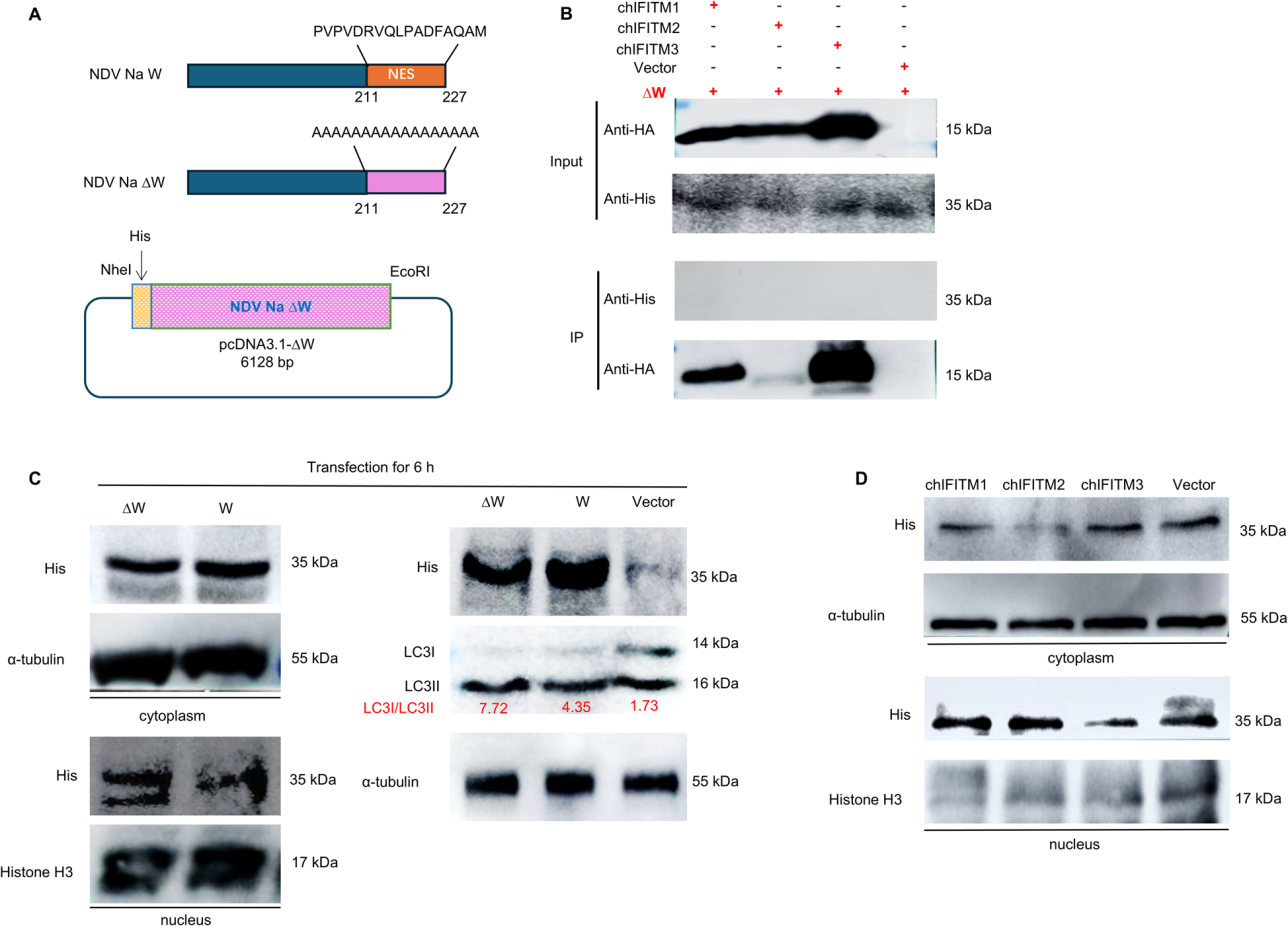
**Preliminary study on the mechanism by which chIFITMs affect the action of W proteins.** **A** Schematic diagram of W protein NES mutant construction. **B** Co-IP analysis of the interaction between chIFITMs and ∆W. **C** The cellular localization of W and ∆W after transient overexpression for 6 h and the effect on the level of the autophagy protein LC3 were determined via His/LC3/α-tubulin/histone H3 primary antibodies and HRP-conjugated goat anti-rabbit/mouse IgG (H + L). **D** Effect of chIFITM overexpression on the localization of the W protein.

In the early stage, we co-transfected IFITMs and NDV W via transient co-transfection and confirmed their interaction by Co-IP (Figure 7E). When consulting the literature, we found that the NDV W protein has different cellular localizations in different periods: 2–4 h in the nucleus and 6–8 h in the cytoplasm, and the nuclear export-signal (NES) domain determines the cellular localization of the W protein [36]. Therefore, we propose the following hypothesis: is autophagy triggered by the mislocalization of the W protein after the interaction occurs? Therefore, we constructed mutants in the NES region of the W protein (∆W) and found that the interaction between IFITMs and the W protein disappeared, suggesting that the interaction between IFITMs and W depends on the NES region (Figures 8A, B). We detected the cellular localization and LC3II level after overexpression of the wild-type W protein or the mutant w protein (∆W) and found that the cellular localization of the mutant protein changed from the cytoplasm to the nucleus, increasing the level of autophagy (Figure 8C). The overexpression of IFITM1/2 affected the cellular localization of the W protein, whereas IFITM3 did not affect W localization, suggesting that IFITM3 does play a role through a pathway different from that of IFITM1/2 (Figure 8D).

## Discussion

IFITMs, recognized as typical interferon-induced effectors, have been extensively studied since their antiviral effects were identified across various species, including humans, mice, and pigs [50]. They have demonstrated efficacy in limiting infections caused by diverse viruses, including major pathogens from the families *Orthomyxoviridae*, *Filoviridae*, *Coronaviridae*, *Retroviridae*, and *Flaviviridae*, which pose significant threats to human health and societal stability [51–59]. While research on human IFITMs is comprehensive, encompassing gene evolution, structural analysis, critical amino acids, and antiviral mechanisms, studies on avian IFITMs are limited and have focused primarily on sequence analysis, genetic evolution, and phenotypic identification [10, 13, 46, 60–64].

This study delves into the gene loci, antiviral activity, and mechanisms of action of chIFITMs. Sequence analysis revealed that chIFITMs share similar loci and structures with IFITMs from other species, featuring an N-terminal domain (NTD), an intracellular medial domain (IMD), a conserved intracellular loop (CIL), a transmembrane domain (TMD), and a C-terminal domain (CTD). Multiple conserved sites, such as Y20 [47], C71/72 [48], GxxxG [6], and YxxΦ [49], further emphasize the functional similarity between chIFITMs and other species. Antiviral experiments demonstrated that chIFITMs regulate cell membrane fluidity, inhibit virus entry into cells, and effectively curb NDV and IAV replication [65].

Paramyxoviruses, such as Nipah virus (NiV), parainfluenza virus type 3 (PIV3), and NDV, can cause many important infectious diseases that negatively impact both human and animal health. NDV causes significant disease in most bird species, imposing a heavy burden on both agriculture and the economy [36].

Viral particles of members of the *Paramyxoviridae* family contain a single molecule of linear, negative-sense, single-stranded RNA that encodes 6 to 10 proteins, some of which can be derived from the P gene through RNA editing events. Through these events, V and W mRNAs can be generated by inserting pseudotemplated G nucleotides into conserved sites [66]. The V protein and W protein share the same N-terminal structural domain but different C-terminal structural domains, and the V protein is localized in the cytoplasm and is an important virulence factor and IFN antagonist of the *Paramyxoviridae* family, which has highly conserved cysteine-rich CTDs that can be used through multiple strategies to bypass the host IFN pathway [67]. However, there is limited information on the editing product W protein, mostly from studies of NiV, which has a 1:1:1 ratio of P:V:W mRNA for the P gene transcript, and the ratio of W protein is significantly greater in NiV than in other viruses [68]. The W protein of NiV, which can be detected in the nucleus of infected cells, exerts anti-IFN effects by inhibiting the JAK/STAT signalling pathway and the toll-like receptor 3 (TLR3) pathway. Previous studies have shown that the W protein can be detected in NDV-infected cells and enhances NDV replication and increases the viral titre. Furthermore, interference with IFITMs resulted in decreased antiviral activity of chicken interferon lambda 3 (chIFNL3), highlighting the crucial role of IFITMs in the interferon pathway [69].

This study also introduced and validated the hypothesis of an association between chIFITMs and the NDV W protein, an important virulence factor. The overexpression of chIFITMs reduced W protein expression, and 3-MA treatment indicated that chIFITM1 and chIFITM2 may degrade W protein through the autophagy pathway. The Co-IP results suggested a direct interaction between chIFITMs and W protein, indicating that their inhibitory effect may involve direct interaction with and activation of the autophagy pathway. We validated the antiviral phenotype of IFITMs via both strong and weak strains of NDV and conducted mechanistic studies on the strong strain Na. Although in vitro experiments confirmed the antiviral results of chIFITMs, in vivo experiments were not performed for validation because of the lack of animal models and laboratory biosafety requirements. At present, we only initially explored the relationship between IFITMs and NDV W proteins, and we will follow up with an in-depth study of the structures and sites, analyse their interactions, and analyse in depth the generic and unique mechanisms by which IFITMs function.

In conclusion, this study provides a comprehensive analysis of the antiviral effects and mechanisms of chIFITMs, offering valuable insights for future research on avian IFITMs. Although IFITMs have excellent antiviral effects, many difficulties remain in their practical application. For example, IFITMs are transmembrane proteins (molecular weights of approximately 15–20 kDa) that need to be localized to the cell membrane or endosomal membrane to function. Direct delivery of intact proteins has difficulty crossing the cell membrane barrier. At present, existing delivery systems (such as liposomes and nanoparticles) also have difficulty accurately locating specific subcellular regions (such as the endosomal membrane). The specific roles of the N-terminal domain, transmembrane region and C-terminal intracellular region of IFITMs in antivirals have not been fully resolved, making it difficult to design simplified small-molecule mimics. The widely proven mechanism of action of IFITMs involves changing the physical properties of the membrane. Its dynamic conformation is difficult to capture by traditional structural biology methods, hindering drug design. Moreover, IFITMs regulate the characteristics of the cell membrane to inhibit viruses, which may interfere with normal cell functions (such as membrane fluidity and cholesterol metabolism), leading to off-target toxicity. Exogenous IFITMs may be recognized by the immune system as antigens, triggering antibody neutralization or allergic reactions; in particular, when IFITMs are repeatedly administered, the risk is greater. There is a lack of biomarkers for evaluating the activity of IFITMs in vivo, and it is difficult to quantify their efficacy or optimize the dose in clinical trials. In general, IFITMs, as natural antiviral proteins, have the potential for broad-spectrum inhibition of viral invasion, but their drug formation faces multiple challenges, such as protein stability and delivery difficulties, structural complexity, and difficulties in clinical transformation.

## Supplementary Information

**Additional file 1: Co-IP analysis of the interaction between endogenous chIFITM3 and NDV W protein.**

**Additional file 2: Establishment of cell infection models with different strains.** Different viral multiplicities of infection and infection times were set. CCK8 was used to detect the effect of viral infection on cell viability, and the multiplicity of infection without a significant effect on cell viability was selected. The virus proliferation levels of the fluorescent viruses NDV Na EGFP (A), NDV rL EGFP (B), and VSV EGFP (C) were detected via flow cytometry, and H9N2 (D) was detected by a virus titre assay.

**Additional file 3: Western blot was used to detect the effect of siIFITM3 knockdown.**

## Data Availability

All data generated or analyzed during this study are included in this published article.
