# Supplementary figures and images for: Chicken interferon-induced transmembrane proteins inhibit Newcastle disease virus infection by affecting viral entry and W protein expression
Source: Vet Res. 2025 May 21;56:104. doi: 10.1186/s13567-025-01530-y (PMC12093685; doi:10.1186/s13567-025-01530-y)

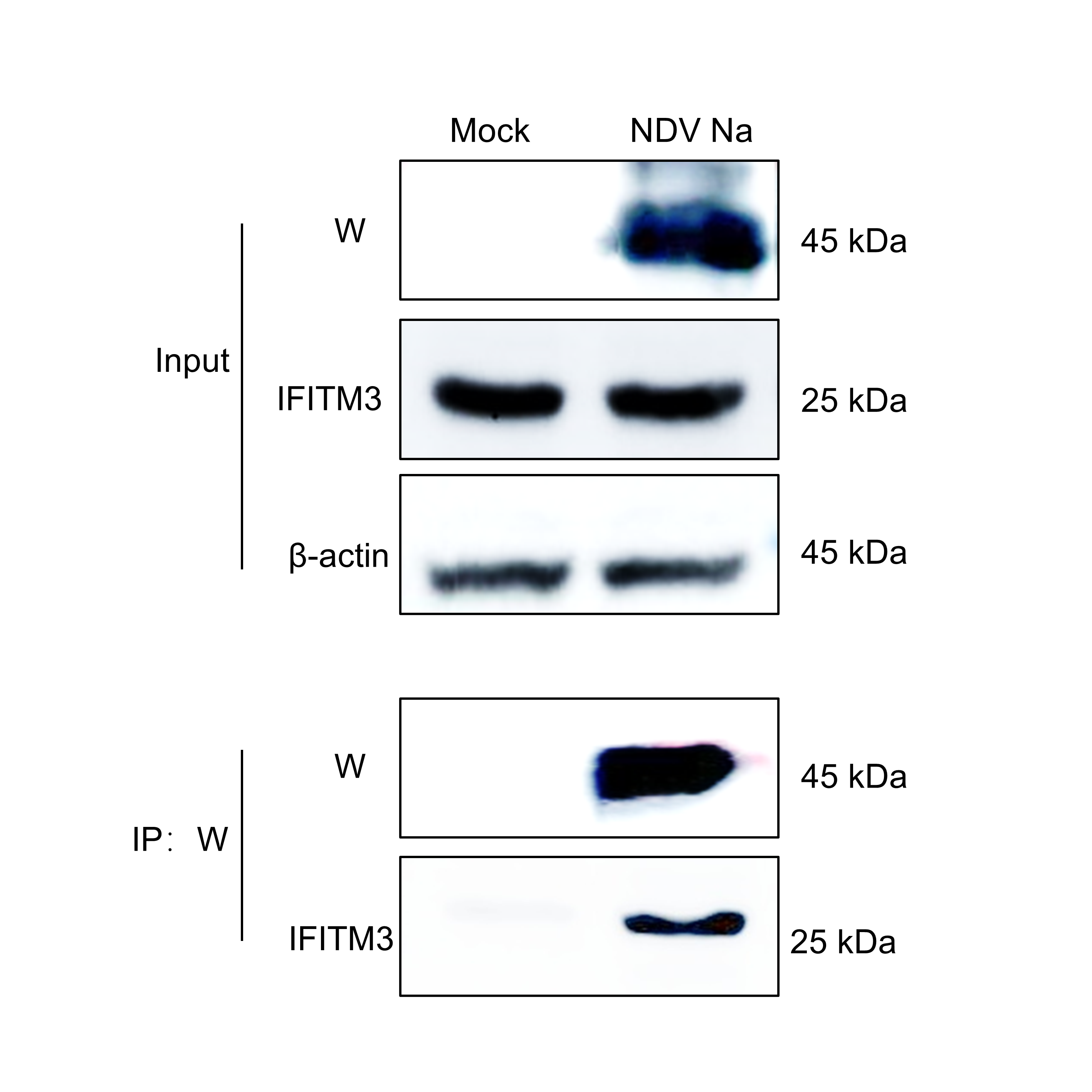

Supplement: Supplementary file 1 — Additional file 1: Co-IP analysis of the interaction between endogenous chIFITM3 and NDV W protein. [file 13567_2025_1530_MOESM1_ESM.tif]

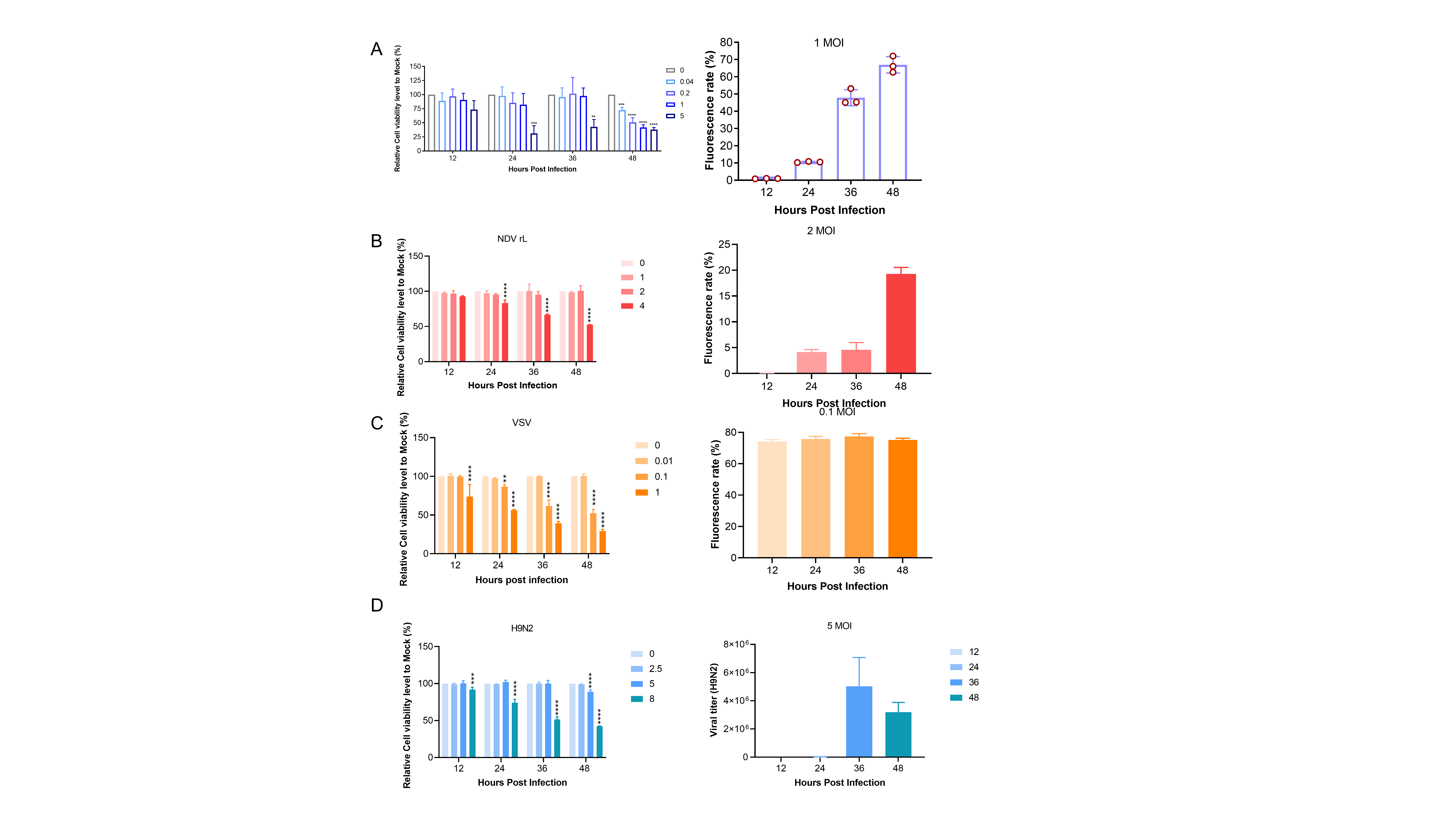

Supplement: Supplementary file 2 — Additional file 2: Establishment of cell infection models with different strains. Different viral multiplicities of infection and infection times were set. CCK8 was used to detect the effect of viral infection on cell viability, and the multiplicity of infection without a significant effect on cell viability was selected. The virus proliferation levels of the fluorescent viruses NDV Na EGFP (A), NDV rL EGFP (B), and VSV EGFP (C) were detected via flow cytometry, and H9N2 (D) was detected by a virus titre assay. [file 13567_2025_1530_MOESM2_ESM.tif]

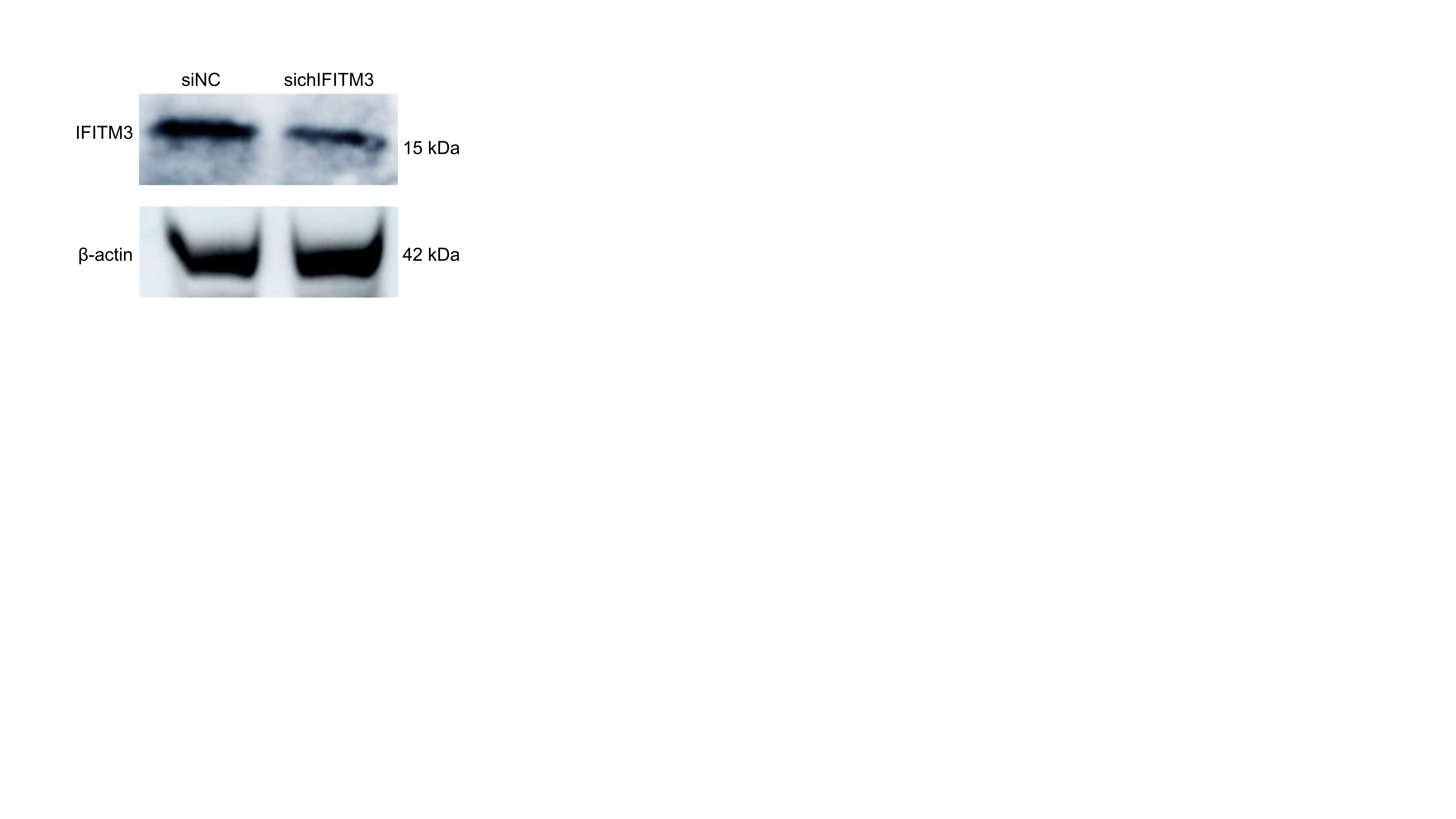

Supplement: Supplementary file 3 — Additional file 3: Western blot was used to detect the effect of siIFITM3 knockdown. [file 13567_2025_1530_MOESM3_ESM.tif]
